# Peripheral Blood Mitochondrial DNA Levels Were Modulated by SARS-CoV-2 Infection Severity and Its Lessening Was Associated With Mortality Among Hospitalized Patients With COVID-19

**DOI:** 10.3389/fcimb.2021.754708

**Published:** 2021-12-16

**Authors:** José J. Valdés-Aguayo, Idalia Garza-Veloz, José R. Vargas-Rodríguez, María C. Martinez-Vazquez, Lorena Avila-Carrasco, Sofia Bernal-Silva, Carolina González-Fuentes, Andreu Comas-García, Diana E. Alvarado-Hernández, Alba S. H. Centeno-Ramirez, Iram P. Rodriguez-Sánchez, Ivan Delgado-Enciso, Margarita L. Martinez-Fierro

**Affiliations:** ^1^ Molecular Medicine Laboratory, Unidad Académica de Medicina Humana y C.S, Universidad Autónoma de Zacatecas, Zacatecas, Mexico; ^2^ Departamento de Microbiología, Facultad de Medicina, Universidad Autónoma de San Luis Potosí, San Luis Potosí, Mexico; ^3^ Centro de Investigación en Ciencias de la Salud y Biomedicina, Universidad Autónoma de San Luis Potosí, San Luis Potosí, Mexico; ^4^ Service of Internal Medicine, Hospital General de Zacatecas “Luz González Cosío”, Zacatecas, Mexico; ^5^ Public Health Laboratory, Zacatecas State Health Services, Zacatecas, Mexico; ^6^ Facultad de Ciencias Biológicas, Laboratorio de Fisiología Molecular y Estructural, Universidad Autónoma de Nuevo León, Nuevo León, Mexico; ^7^ Facultad de Medicina, Universidad de Colima, Colima, Mexico

**Keywords:** mitochondrial DNA, mitochondria, severity, COVID-19, SARS-CoV-2

## Abstract

**Introduction:**

During severe acute respiratory syndrome coronavirus 2 (SARS-CoV-2) infection, the virus hijacks the mitochondria causing damage of its membrane and release of mt-DNA into the circulation which can trigger innate immunity and generate an inflammatory state. In this study, we explored the importance of peripheral blood mt-DNA as an early predictor of evolution in patients with COVID-19 and to evaluate the association between the concentration of mt-DNA and the severity of the disease and the patient’s outcome.

**Methods:**

A total 102 patients (51 COVID-19 cases and 51 controls) were included in the study. mt-DNA obtained from peripheral blood was quantified by qRT-PCR using the NADH mitochondrial gene.

**Results:**

There were differences in peripheral blood mt-DNA between patients with COVID-19 (4.25 ng/μl ± 0.30) and controls (3.3 ng/μl ± 0.16) (*p* = 0.007). Lower mt-DNA concentrations were observed in patients with severe COVID-19 when compared with mild (*p*= 0.005) and moderate (*p*= 0.011) cases of COVID-19. In comparison with patients with severe COVID-19 who survived (3.74 ± 0.26 ng/μl) decreased levels of mt-DNA in patients with severe COVID-19 who died (2.4 ± 0.65 ng/μl) were also observed (*p* = 0.037).

**Conclusion:**

High levels of mt-DNA were associated with COVID-19 and its decrease could be used as a potential biomarker to establish a prognosis of severity and mortality of patients with COVID-19.

## Introduction

Severe acute respiratory syndrome coronavirus 2 (SARS-CoV-2) was identified for the first time in January 2020 (Cheng et al., 2020), and it is the infectious agent that causes coronavirus disease 2019 (COVID-19). Worldwide, as of November 2021, there have been approximately 253,312,354 confirmed cases of COVID 19, including 5,100,228 deaths reported (Dong et al., 2020).

SARS-CoV-2 consists of a genome made up of positively charged RNA (Zhou et al., 2020), and a petal-shaped projection called a spike protein that mediates the binding of the virus and membrane fusion to the surface receptor for angiotensin converting enzyme 2 (ACE-2) in host cells during infection (King et al., 2012). SARS-CoV-2 infection causes an inflammatory process that promotes the release of multiple pro-inflammatory cytokines which recruits circulatory leukocytes and amplifies inflammation (Hamming et al., 2008). The hyper-inflammatory state induced by COVID-19 and the subsequent oxidative stress (OS) events are closely related to the mitochondrion (Shenoy, 2020), an organelle whose function is to regulate multiple cell metabolism pathways and produce energy by the assembly of adenosine triphosphate (ATP) *via* oxidative phosphorylation (OXPHOS) (Singer, 2014). The mitochondrion contain its own circular genome called mitochondrial DNA (mt-DNA), which is replicated independently of the host genome, and encodes 13 polypeptides responsible for OXPHOS (Annesley and Fisher, 2019; Hoffmann et al., 2020). Lack of histones in mt-DNA (Nunnari and Suomalainen, 2012) and its close proximity to the site of reactive oxygen species (ROS) production in the mitochondrial membrane, makes it vulnerable to damage, especially to OS caused by inflammatory states, reducing the capacity of mt-DNA repair and increasing its susceptibility to mutations and deletions (Sharma and Sampath, 2019).

When SARS-CoV-2 enters the cell in the cytoplasm the virus needs to initiate replication through an intermediate molecule, double stranded RNA (dsRNA) (Sethna et al., 1989). This process predisposes the detection of the virus by pathogen recognition receptors (PRRs) and mitochondrial viral signaling systems (Knoops et al., 2008). SARS-CoV-2 evades this detection by inducing the production of double membrane vesicles with the help of mitochondria and the membranes of the endoplasmic reticulum (ER), achieving successful replication and dissemination (Hagemeijer et al., 2012; Valdes-Aguayo et al., 2021). However, the processes related to the viral replication, dissemination, and virus immune cell evasion may damage the membranes of mitochondria and the mt-DNA genome, causing the leakage of altered mt-DNA into the cytoplasm. These released mt-DNA fragments act as danger-associated molecular patterns (DAMPs) (Faas and de Vos, 2020), which are identified by PRRs, such as toll like receptors (TLRs), that activates the nuclear factor kappa-light-chain-enhancer of activated B cells (NF-Kβ) pathway and the pyrin domain-containing protein 3 (NLRP3) inflammasome, triggering a pro-inflammatory response with the release of pro-inflammatory cytokines, such as TNF-α, IL-1, IL-6, and IL-8 which are associated with the exacerbation of the inflammatory response of COVID-19 (Nakahira et al., 2013).

The alteration in mt-DNA quantity and quality as a biomarker of disease has been reported in chronic diseases, such as obesity, insulin resistance, neurodegenerative conditions, and cardiovascular disease (Yue et al., 2018; Lowes et al., 2020; Morandi et al., 2020; Sharma et al., 2021). Despite the presence of these pathologies being demonstrated to increase the risk of severe disease and/or death when they are present in patients with COVID-19, the relation between levels of mt-DNA and COVID-19 has scarcely been studied. However, growing evidence indicate that the release of damaged mt-DNA is related to the increase of the systemic inflammatory response, allowing us to think of mt-DNA as a potential non-invasive tool that may be useful to predict the evolution and the selection of a successful approach in a patient with COVID-19. According to the above, in this study, we explored the importance of peripheral blood mt-DNA as an early predictor of evolution in hospitalized and non-hospitalized patients with COVID-19. The association between the concentration of mt-DNA, the severity of the disease and the patient’s outcome was also explored.

## Methods

### Study Population

This transversal study was approved by the Research Committee of the Academic Unit of Human Medicine and Health Sciences from Universidad Autónoma de Zacatecas *“Francisco García Salinas”* and the Ethic and Investigation Committee of the Hospital General de Zacatecas *“Luz González Cosío”* (ID number: 0223/2021/C), in Zacatecas Mexico. Patients who authorized their participation in this protocol, through the signing of the informed consent, were included in the study. Inclusion criteria for the study included patients older than 18 years with indicated screening for SARS-CoV-2. Later, patients were classified as cases (COVID-19 infection confirmed by positive RT-PCR test) or controls (patients with a negative RT-PCR result for SARS-CoV-2). COVID-19 positive patients with moderate and/or severe (see below the classification criteria) who met the hospitalization standards established by the Ministry of Health of Zacatecas (Información, 2020) and required hospitalization at the Hospital General de Zacatecas *“Luz González Cosío”* where included in the study. The recruitment of patients with mild COVID-19 and controls was carried out in the facilities of Molecular Medicine Laboratory at Universidad Autónoma de Zacatecas. According to the above, a total of 102 participants were recruited consecutively: 51 COVID-19 cases and 51 controls.

### Clinical Information and Criteria for the Classification of Patients According to the Severity of COVID-19

On the day of hospital admission, each patient answered a standardized questionnaire inquiring about medical history, lifestyle, and intake medications. To classify their severity, trained health personnel measured the following clinical parameters: anthropometric characteristics, temperature, heart and respiratory rates, blood pressure and oxygen saturation (SaPO_2_). Laboratory data were taken from the clinical records, and they included the following: hemoglobin, urea, blood urea nitrogen (BUN), glucose, serum electrolytes, albumin, coagulation tests, C-reactive protein (CRP), procalcitonin, creatine kinase (CK), ferritin, lactate dehydrogenase (LDH) and direct bilirubin.

On the other hand, after their inclusion in the study, patients with COVID-19 were classified as mild, moderate or severe according to the guidelines of the Mexican Secretary of Health and the Instituto Mexicano del Seguro Social (IMSS) (Secretaría de Salud, 2020), in addition to clinical scales such as the Sequential Organ Failure Assessment (SOFA) score and Confusion, Urea nitrogen, Respiratory rate, Blood pressure, 65 years of age and older (CURB-65), and specialized computerized scales, such as ABC-GOALS and the COVID-GRAM Critical illness risk score, in which clinical and laboratory parameters were analyzed (Liang et al., 2020; Mejia-Vilet et al., 2020). We defined a severe COVID-19 case when the patient presented with CURB-65 ≥ 3, SOFA score ≥ 3, ABC-GOALS ≥ 10 points or a COVID-GRAM Critical illness risk score ≥ 40.4%. A moderate COVID-19 case was determined by having a CURB-65 ≤ 2, SOFA score ≤ 3, ABC-GOALS between 4 and 10 points or a COVID-GRAM Critical illness risk score from 1.7 to 40.3%. Finally, a patient with a mild case of COVID-19 was defined as having a CURB-65 between 0 and 1 point, ABC-GOALS between 0 and 3 points or a COVID-GRAM Critical illness risk score < 1.7%. The SOFA score was not used to patients with mild disease, because they met criteria for neither hospitalization nor admission to the intensive care unit (ICU).

### Biological Samples and DNA Isolation From Peripheral Blood Samples

During the patient admission, 2 ml of peripheral blood were obtained in a standard tube with anticoagulant (EDTA). After collection, all peripheral blood samples were immediately processed for DNA isolation. DNA from the patients’ samples was obtained from whole blood. A total 100 μl of peripheral blood were used to obtain total genomic DNA using a standard phenol/chloroform technique. The concentration of isolated DNA was measured on a NanoDrop-1000 spectrophotometer (Thermo Scientific, Waltham, MA USA). The DNA samples were stored at -20°C until use.

### Obtaining Mitochondria and mt-DNA Isolation

Mitochondria from human peripheral blood mononuclear cells were extracted to quantify the number of copies of mt-DNA and to establish a range of mt-DNA detection as it is explained in the next section. We used a MEB extraction buffer that consisted of 0.25 M sucrose, 20 mM HEPES-KOH, pH 7.5, 10 mM KCl, 1.5 mM MgCl_2_, 1 mM EDTA, 1 mM EGTA, 1 mM dithiothreitol and 0.1 mM PMSF. Mitochondria were isolated using the method described by Pin-Chao Liao et al. (2020). After mt-DNA was isolated as mentioned in the previous section, it was stored at -20°C until use.

### Measurement of mt-DNA by qRT-PCR

To determine the mt-DNA content (ng/μl), we used quantitative real-time polymerase chain reaction (qRT-PCR). All qRT-PCR reactions were carried out in 10 μl of reaction, using 1X SYBR Green (Thermo Scientific) reagent and the 200 nM of the following forward and reverse primers: 5’-ATACCCATGGCCAACCTCCTAC-3’ and 5’-GGGCCTTTGCGTAGTTGTATATA-3’, which amplify a fragment of 110 bp of the nicotinamide adenine dinucleotide (NADH) gene. The qRT-PCR thermal conditions were 95°C for 15 minutes, followed by 40 cycles at 95°C for 15 s, 60°C for 30 s and 72°C for 30 s. Melting curve analysis of 95°C for 15 s, 60°C for 15 s and 95°C for 15 s at the end of each run was added to confirm the specificity of the PCR products. qRT-PCR was carried out in a StepOne Plus Real-Time PCR Systems analyzer (Applied Biosystems).

In a first step to identify the dynamic range of the method selected for mt-DNA quantification, we performed two standard curves of concentrations: one of them using total DNA of a pooled sample from 10 control subjects and the other using mt-DNA isolated from peripheral mononuclear cells. Both curves we carried out in triplicate using logarithmic dilutions from 100 ng to 0.001 ng. Afterwards, we included 20 individual DNA samples from 20 patients randomly selected included to guarantee that the Cq value showed amplification inside the range of dilutions selected. In this analysis, 10 ng of total DNA were included. The quantification of the number of copies of mt-DNA was obtained from the second curve (mt-DNA) using the formula: [Amount (ng) x 6.022 x 10^23^]/[Length (bp) x (1 x 10^9^) x 660] and they were used for the establishment of the dynamic range of detection of the method evaluated regarding the number copies of mt-DNA (lower and upper detection limits). In a second stage, to quantify the mt-DNA from the peripheral blood samples of the individuals included in the study, 10 ng of total DNA from whole blood were used in each reaction plate. The qRT-PCR and thermal conditions were as described previously. In all the reaction plates, a standard curve consisting of mt-DNA dilutions from 100 ng to 0.001 ng were included.

For consistency, all samples were run in duplicate, and two non-template controls were included in each assay. The coefficient of variation between duplicate measurements within the same run was < 1% for each of the amplified sequences, and < 5% for the inter-run samples.

### Statistical Methods

Data organization and log transformation were done using Microsoft Excel Software for statistical analyses. Chi-squared and Student’s t tests were used to determine changes between study groups. The Pearson correlation coefficient was used to evaluate the relationship between mt-DNA and clinical variables. To identify cutoff values of mt-DNA associated with COVID-19 outcomes the area under the curve (AUC) was obtained using the receiver operating characteristics (ROC) curve analysis. The score cutoff that maximized Youden’s index in the training set was used to estimate sensitivity and specificity. All statistical analysis was done using *SigmaPlot v12.0 software* (Systat Software Inc., San Jose, CA). Statistical significance was considered with a two-sided significance level of < 0.05.

## Results

### Characteristics of the Study Population

The study population consisted of 102 participants, 51 COVID-19 positive patients and 51 controls. The demographic and clinical characteristics of the COVID-19 positive cases and controls are summarized in **Table 1**. The median age of the COVID-19 positive cases was 47.7 years (range: 64.3-31.3 years), whereas the average age of the control patients was 43.8 years (range: 54.7- 32.9 years). Thirty (58.8%) COVID-19 positive patients and 36 (70.5%) control patients were men. Among the comorbidities in patients with COVID-19, 10 (19.6%) patients had type 2 diabetes mellitus (T2DM), 15 (29.41%) patients had obesity, 18 (35.29%) patients had arterial hypertension and 18 (35.2%) patients suffered from other pathologies, such as chronic obstructive pulmonary disease (COPD), cardiovascular disease, immunosuppression, and anemia. Otherwise, in the control group, 7.8% of the patients had T2DM, and 9.8% of the patients had obesity. Considering the controls as reference, the COVID-19 cases group had a higher proportion of patients with arterial hypertension and obesity (*p* < 0.05). Among patients with severe COVID-19 and the control group, obesity was a factor found in 12 (48%) and 5 (9.8%) patients, respectively (*p ≤* 0.001). There were differences in BMI values (kg/m^2^) between patients with severe COVID-19 and the control group [OR = 8.5, 95% confidence interval (95% CI): 1.82 – 5.48; *p* ≤ 0.001]. Furthermore, statistical difference was reached in SaPO_2_ (%) parameters between the control group and patients with severe COVID-19 (*p ≤* 0.001). No additional differences regarding other general characteristics or risk factors between groups were identified (*p* > 0.05).

**Table 1:** General characteristics of the study population classified as control group and COVID-19 positive cases.

Characteristics	COVID-19 (n=51)	Controls (n=51)	p-value	OR
				(95% CI)
Sex n (%)				
Male	30 (58.82)	36 (70.58)	0.3	0.6 (0.3-1.4)
Female	21 (41.17)	15 (29.41)		
Age (years)	47.74 ± 16.68	43.8 ± 10.9	0.163	
Occupation (%)				
Housewives	9 (36)	1 (1.96)	0.397	N/A
Health workers	6 (24)	18 (35.29)		
Administrative personnel	22 (43.13)	18 (35.29)		
Farmers	5 (20)	3 (5.88)		
Military	3 (12)	0		
Engineers	3 (12)	7 (13.7)		
Miners	3 (12)	4 (7.8)		
Comorbidities n (%)				
Type 2 diabetes mellitus	10 (19.60)	4 (7.8)	0.15	2.9 (0.8-9.8)
Obesity	15 (29.41)	5 (9.8)	0.025*	3.8 (1.3-11.5)
Arterial hypertension	18 (35.29)	0	<0.001*	N/M
Others	18 (35.2)	3 (5.8)	<0.001*	8.7 (2.4-32)
Addictions n (%)				
Smoking	9 (17.64)	8 (15.68)	1	1.2 (0.4-3.3)
Other parameters (mean ± SD)				
SaPO_2_	86.9 ± 11.3	93.9 ± 2.2	<0.001*	N/A
BMI (Kg/m^2^)	28.7 ± 5.6	25.1 ± 3.5	<0.001*	N/A
Cq value (SARS-CoV-2 N gene)	25.3 ± 7.3	N/A	N/A	N/A
mt-DNA (ng/μl)	4.3 ± 2.2	3.3 ± 1.1	0.007*	N/A

Data are presented as frequency and percentages. SD, standard deviation; BMI, body mass index; SaPO_2_, oxygen saturation; N/A, not applicable. N/M, not measurable. *p <0.05.

Regarding the patients classified as having mild, moderate and severe COVID-19, the mean age was of 44.5 ± 13.8 years, 43.1 ± 10.3 years and 51.6 ± 19.7 years respectively (p > 0.05). With regard to the sex of the patients, there were more male than female patients in the three groups, with 9 (56.2%) patients in the mild group, 8 (80%) patients in the moderate group and 13 (52%) patients in the severe group. Obesity, T2DM and arterial hypertension were the comorbidities most relevant in the three groups. In relation to obesity, 2 (12.5%), 1(10%) and 12 (48%) patients had this comorbidity in the mild, moderate and severe groups respectively. In relation to T2DM, there was 1(10%) patients in the moderate group, and 9 (36%) patients in the severe group. Furthermore, arterial hypertension was a significant disease in the severe group, with 15 (60%) patients and in the moderate group, with 3 (30%) patients. Regarding the Cq values of the N gene of SARS-CoV-2 in patients with mild (26.16 ± 7.94), moderate (23.83 ± 7.55) and severe COVID-19 (25.48 ± 6.8), no differences were found. No additional differences were found among general characteristics in groups of patients with mild, moderate and severe COVID-19.

### Mitochondrial DNA Quantification

**Figure 1:**
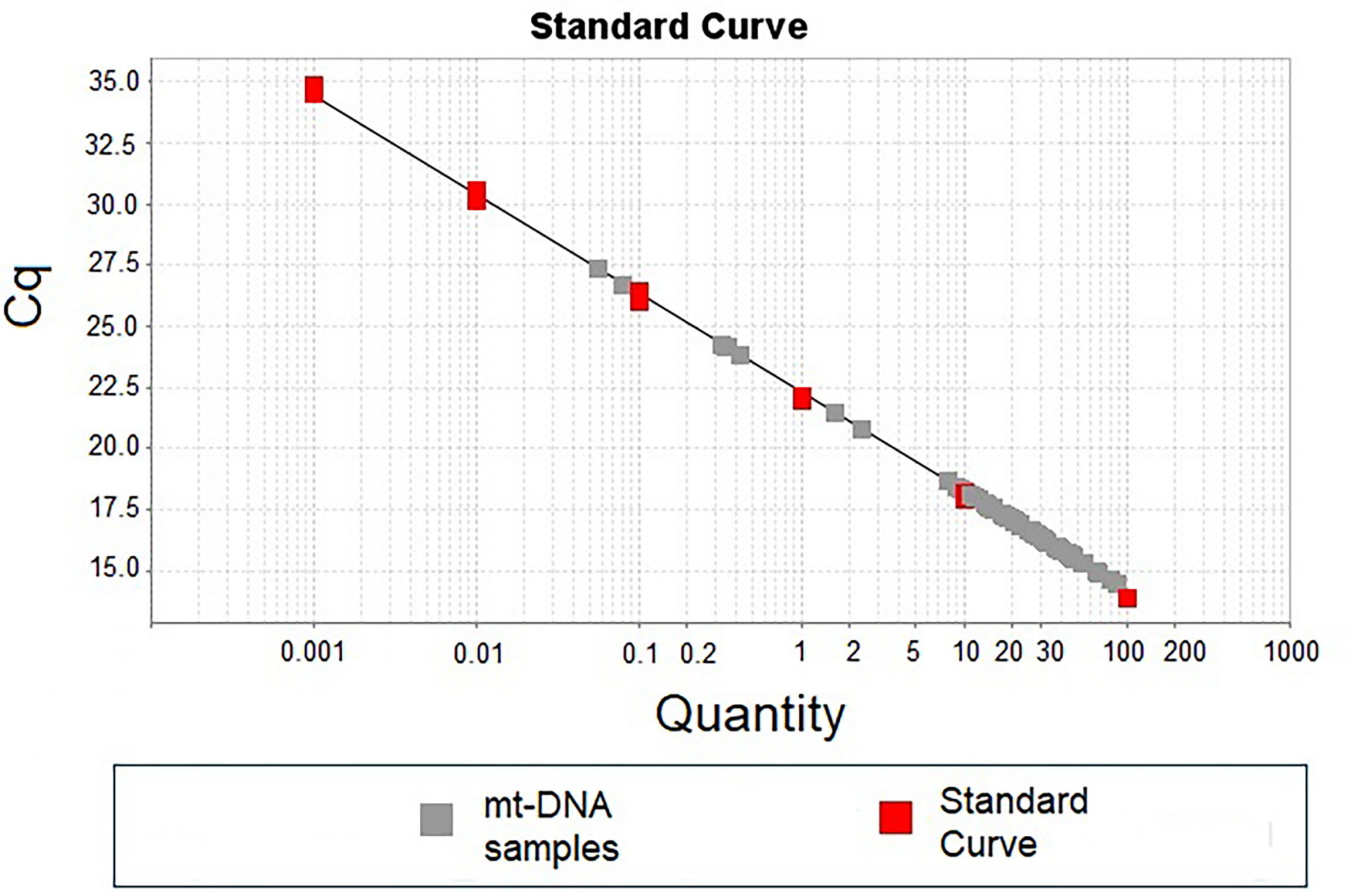
Standard curve of mt-DNA concentration using. Standard curve was carried out using logarithmic mt-DNA concentrations from 0.01 ng to 100 ng (red dots in the plot). A total of 10 ng of DNA from each patient was used to detect the number of copies of mt-DNA (gray dots in the plot).

**Figure 1** shows a representative standard curve obtained during the first stage of standardization of the mt-DNA quantification, which was aimed to identify the dynamic range of detection.

Our results showed that quantities lower than 0.01 ng of total DNA showed no qRT-PCR amplification of the mt-DNA region selected. In the same way and because the Cq of all the individuals included in the standardization stage were inside the points of the standard curve, 10 ng of total DNA from each patient was included in all the determinations (**Figure 1**).

The dynamic range of detection of the method evaluated regarding number copies of mt-DNA was from 5.59 × 10^4^ (lower detection limit evaluated) to 5.59 × 10^9^ (upper detection limit evaluated) copies. During the stage of quantification of mt-DNA, the mean of mt-DNA concentrations for the patients with COVID-19 was 4.25 (range: 0.31-13.35) and of 3.3 (range: 0.82 - 5.75) for the controls (*p* < 0.05) (**Table 1**).

### Correlations Between mt-DNA and Clinical Features of Patients With COVID-19

To evaluate the relation between peripheral blood mt-DNA and the clinical features of the patients with COVID-19, correlation tests were carried out. These features included BMI, temperature, heart and respiratory rates, blood pressure, SaPO_2_ and laboratory measurements (**Tables 2**–**4**). The results of the correlation tests are shown in **Tables 2**, **3**. Parameters such as mean corpuscular hemoglobin, partial thromboplastin time and systolic blood pressure (SBP) correlated positively with the concentration of mt-DNA (*p* < 0.05). The BMI measurements of COVID-19 cases and their peripheral mt-DNA concentration had an inverse correlation (r: -0.645; *p* = 0.012). All the variables with statistical significance identified in the correlation tests are displayed in **Tables 2**, **3**.

**Table 2:** Correlation analysis between laboratory findings and whole blood mt-DNA of severe COVID-19 patients.

Variable 1	Variable 2	Correlation coefficient	*p*-value
mt-DNA	MCH	0.595	0.019
	RDW-CV	0.557	0.031
	RDW-SD	0.649	0.008
	PTT	0.562	0.036
Erythrocytes	Hb	0.962	<0.001
	HCT	0.976	<0.001
	MCV	-0.643	0.009
Hb	HCT	0.992	<0.001
VGM	MCH	0.554	0.031
	RDW-SD	0.696	0.003
	Na^+^	-0.557	0.038
MCH	PT	-0.668	0.009
	PTT	0.574	0.0317
RDW-CV	RDW-SD	0.886	<0.001
Platelets	Lymphocytes	0.753	0.001
Leucocytes	Neutrophils	0.986	<0.001
RDW-SD	PTT	0.659	0.01
Monocytes	Urea	0.724	0.018
	BUN	0.72	0.028
Eosinophils	Cl^+^	0.634	0.036
Basophils	Globulin	0.652	0.04
Glucose	Creatinine	0.568	0.027
	Ca^2+^	-0.661	0.01
	Mg^2+^	0.739	0.003
	Direct Bilirubin	0.697	0.003
	Fibrinogen	0.597	0.04
Urea	Creatinine	0.723	0.002
	BUN	1	<0.001
Creatinine	BUN	0.753	0.002
	P^+^	0.613	0.034
	Mg^2+^	0.695	0.008
	Ferritin	0.701	0.035
BUN	P^+^	0.613	0.034
	Mg^2+^	0.857	<0.001
Total Proteins	Albumin	0.86	<0.001
	LDH	-0.651	0.015
P^+^	Mg^2+^	0.757	0.002
Ca^2+^	Mg^2+^	-0.701	0.007
	PTT	0.633	0.02
	Fibrinogen	-0.797	0.001
Cl^+^	Na^+^	0.765	0.001
Na^+^	AST	-0.71	0.004
Mg^2+^	Fibrinogen	0.728	0.011
Albumin	CRP	-0.636	0.035
LDH	CK	0.638	0.025
PT	INR	0.738	0.002
PTT	Fibrinogen	-0.624	0.04

AST, aspartate transaminase; BUN, blood urea nitrogen; Ca^2+^, calcium; CK, creatin Kinase; Cl^+^, chlorine; CRP, C-Reactive protein; Hb, hemoglobin; HCT, hematocrit; INR, international normalized ratio; LDH, lactate dehydrogenase; MCH, mean corpuscular hemoglobin; MCV, mean corpuscular volume Mg^2+^, magnesium; Na^+^, sodium; P^+^, phosphorus; PT, prothrombin time; PTT, partial thromboplastin time; RDW-CD, red cell distribution width- coefficient of variation; RDW-SD, red cell distribution width- standard deviation.

**Table 3:** Correlation analysis of vital signs, anthropometric characteristics, and peripheral blood mt-DNA in patients with severe COVID-19.

Variable 1	Variable 2	Correlation coefficient	*p*-value
mt-DNA	BMI	-0.645	0.012
	SBP	-0.51	0.05
HR	RR	0.631	0.011
RR	SaPO_2_	-0.517	0.048
SBP	DBP	0.863	<0.001

BMI, body mass index; DBP, diastolic blood pressure; HR, hearth rate; RR, respiratory rate; SBP, systolic blood pressure; SaPO_2_, oxygen saturation.

**Table 4:** Laboratory parameters obtained of severe COVID-19 patients that required hospitalization.

Clinical and laboratory findings	Hospitalized COVID-19 severe cases		p-value
	Patients who died (n=8)	Recovered patients (n=17)	
Age (years)	60.1 ± 17.1	47.7 ± 20.1	0.145
BMI (kg/m^2^)	34.9 ± 7.7	28.4 ± 3.9	0.005*
SBP (mm/Hg)	120.2 ± 21.8	114.4 ± 14.2	0.547
DBP (mm/Hg)	75 ± 13.1	71.3 ± 10.5	0.578
mt-DNA (ng/ul)	2.4 ± 1.8	3.7 ± 1.1	0.037*
Cq value (SARS-CoV-2 N gene)	22.4 ± 6.5	26.7 ± 6.8	0.298
Invasive ventilation (%)	7 (87.5)	4 (23.5)	0.007*
Length of stay in ICU	14.7 ± 6.7	4.2 ± 3.1	0.012*
Hospital length of stay	13 ± 9.1	7 ± 4.6	0.151
Hematic biometry blood test			
Erythrocytes (10^6^/ul)	4.6 ± 1	5.2 ± 1.3	0.439
Hb (g/dl)	14.4 ± 2.2	15 ± 3.8	0.788
Hct (%)	42.7 ± 7.6	45.1 ± 10.4	0.677
MCV (fl)	93.7 ± 4.4	88.1 ± 6.3	0.127
MCH (pg)	29.4 ± 5.6	29.3 ± 2	0.943
RDW-CD (%)	13.7 ± 2.4	14.3 ± 1	0.487
Platelets (10^3^/ul)	202.1 ± 20.3	232.5 ± 77.9	0.464
Leucocytes (10^3^/ul)	9.8 ± 5.2	8.6 ± 1.8	0.483
RDW-SD (fl)	46.6 ± 8.9	47.7 ± 6.2	0.793
Lymphocytes (10^3^/ul)	0.7 ± 0.1	1.2 ± 0.6	0.172
Monocytes (10^3^/ul)	0.5 ± 0.1	0.6 ± 0.3	0.825
Eosinophils (10^3^/ul)	0.002 ± 0.005	0.05 ± 0.1	0.412
Basophils (10^3^/ul)	0.02 ± 0.01	0.03 ± 0.03	0.460
Neutrophils (10^3^/ul)	8.4 ± 5.2	6.9 ± 1.8	0.421
Blood chemistry test			
Glucose (mg/dl)	111.7 ± 34.9	110.4 ± 36.8	0.952
Urea (mg/dl)	35.9 ± 7.2	42.2 ± 26.6	0.654
Creatinine (mg/dl)	0.9 ± 0.06	0.9 ± 0.5	0.832
BUN (mg/dl)	16.7 ± 3.6	16.9 ± 11.3	0.973
Total proteins (g/dl)	6.2 ± 0.6	6.5 ± 0.5	0.393
Serum electrolytes			
P^+^ (mg/dl)	2.9 ± 1.2	3.5 ± 1.3	0.424
Ca^2+^ (mg/dl)	8.2 ± 0.3	8.8 ± 1.2	0.312
Cl^+^ (mmol/l)	99.3 ± 3.5	104.7 ± 3.4	0.018*
K^+^ (mmol/l)	5.5 ± 1.1	4.4 ± 0.7	0.042*
Na^+^ (mmol/l)	133.7 ± 3.2	139.5 ± 2.3	0.004*
Mg^2+^ (mmol/l)	2.3 ± 0.1	2.1 ± 0.5	0.47
Coagulation tests			
PT (seg)	13.6 ± 2	13.8 ± 1	0.793
INR	1.1 ± 0.06	1 ± 0.1	0.285
PTT (seg)	31.2 ± 3.8	34.9 ± 4.8	0.188
Fibrinogen (g/l)	663.2 ± 154.4	550.7 ± 200.7	0.352
Liver function tests			
Total Bilirubin (mg/dl)	0.5 ± 0.1	0.4 ± 0.2	0.098
Direct Bilirubin (mg/dl)	0.2 ± 0.1	0.2 ± 0.1	0.400
Indirect Bilirubin (mg/dl)	0.3 ± 0.1	0.2 ± 0.1	0.056
Albumin (g/dl)	3.3 ± 0.4	3.5 ± 0.5	0.558
ALP (U/l)	95.5 ± 37	120.8 ± 53.1	0.401
ALT (U/l)	31 ± 5.7	40.3 ± 27	0.515
AST (U/l)	52.2 ± 30.9	41.1 ± 18.7	0.416
LDH (U/l)	604.7 ± 231.3	430.3 ± 221.4	0.213
Globulin (g/dl)	2.9 ± 0.4	2.9 ± 0.2	0.605
Inflammation blood tests			
CRP (mg/dl)	16.2 ± 5.4	11.3 ± 6.7	0.311
ESR (mm)	0	29 ± -	N/A
Procalcitonin (ng/ml)	0.8 ± 0.6	0.8 ± 1.1	0.985
D-Dimer (mg/l)	0	812.3 ± -	N/A
Ferritin (ng/ml)	1615.9 ± 117.1	618.4 ± 566.6	0.05*
CK (U/l)	272.3 ± 235.3	90.9 ± 83.1	0.061

Data are showed as mean ± standard deviation. *p <0.05.

ALP, alkaline phosphatase; AST, aspartate transaminase; BUN, blood urea nitrogen; BMI, body mass index; Ca^2+^, calcium; CK, creatin kinase; Cl^+^, chlorine; CRP, C-Reactive protein; DBP, diastolic blood pressure; ESR, erythrocyte sedimentation rate; Hb, hemoglobin; HCT, hematocrit; ICU, intensive care unit; INR, international normalized ratio; LDH, lactate dehydrogenase; MCH, mean corpuscular hemoglobin; MCV, mean corpuscular volume Mg^2+^, magnesium; Na^+^, sodium; P^+^, phosphorus; PT, prothrombin time; PTT, partial thromboplastin time; RDW-CD, red cell distribution width- coefficient of variation; RDW-SD, red cell distribution width- standard deviation; SBP, systolic blood pressure, N/A, Not applicable.

### mt-DNA and COVID-19 Severity

**Figure 2:**
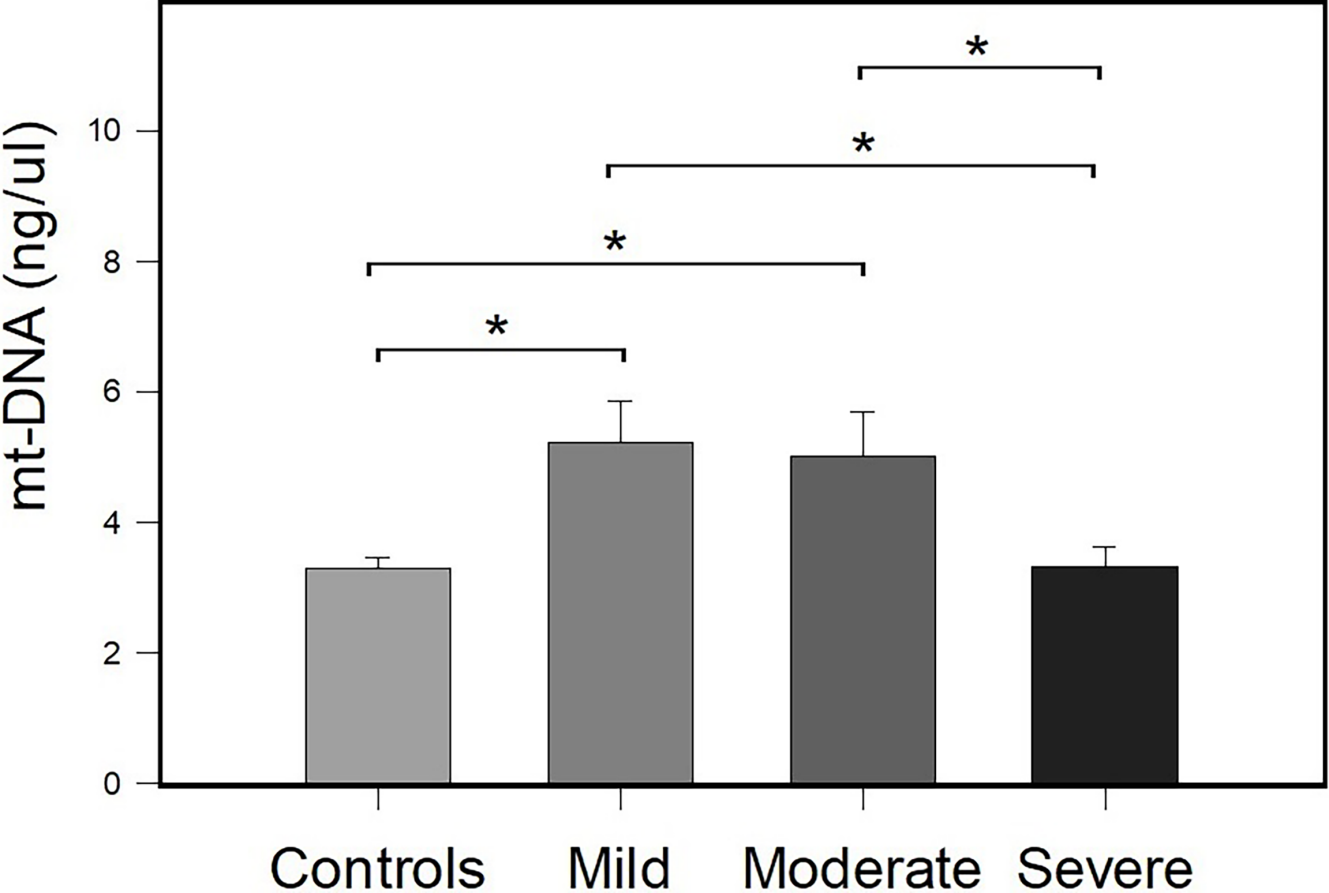
Comparison of the concentration of peripheral mt-DNA in patients with mild, moderate, and severe COVID-19 and the control group. Bar plots represent mean ± standard error. Controls (*n* = 51), and mild *(n* = 16), moderate (*n* = 10), and severe COVID-19 (*n* = 25) cases. *p < 0.05 (Student’s t-test).

To evaluate the association of the peripheral mt-DNA concentration between the control group and patients with COVID-19, the patients with COVID-19 were classified as having mild, moderate, and severe COVID-19 according to their clinical and laboratory parameters (see *Material and Methods* section for details). A total of 25 patients were classified as having severe COVID-19, 10 patients were classified as having moderate COVID-19, and 16 patients were classified as having mild COVID-19. **Figure 2** shows the results of the comparisons obtained in mt-DNA concentrations between the study groups classified according to their severity.

The mean mt-DNA concentrations were 5.22 ± 0.65, 5.01 ± 0.67, 3.32 ± 0.29, and 3.30 ± 0.16, for the groups of patients with mild, moderate and severe COVID-19 and controls, respectively. There were differences in the concentrations of mt-DNA between patients with COVID-19 and controls (*p* = 0.007). Compared with the controls, differences in peripheral mt-DNA levels between patients with mild and moderate COVID-19, reached statistical significance (*p* ≤ 0.001). Differences in mt-DNA concentration between patients with mild COVID-19 and patients with severe COVID-19 (*p* = 0.005), and between patients with moderate COVID-19 and subjects with severe COVID-19 (*p* = 0.011) were also identified. There was a decrease in the amount of mt-DNA in patients with severe COVID-19 in comparison with patients with mild and moderate COVID-19 (**Figure 2**). mt-DNA levels in patients with severe COVID-19 did not reach significant differences when compared with the mt-DNA of control group (*p* = 0.943).

### mt-DNA and Its Association With COVID-19 Outcome

To evaluate if mt-DNA concentrations had differences related with the outcome of COVID-19, the patients hospitalized with severe COVID-19 were stratified according to the reason for their release from the hospital, patients with COVID-19 who died and patients with COVID-19 who recovered. According to their outcomes, 17 patients were released from the hospital due to recovery after improvement in symptoms and laboratory parameters, and 8 patients with severe COVID-19 died after days of hospitalization (**Table 4**). The mean age of patients with severe COVID-19 according to their outcome was of 60.1 ± 17.1 years in the patients who died, and 47.7 ± 20.1 years in the patients with severe disease who survived (p = 0.145). Differences were found when comparing BMI values (kg/m^2^) between patients with severe COVID-19 who died and the patients who recovered (OR= 11; 95% CI: 1.84 – 11.24; *p*= 0.005). In addition, patients who died had a higher frequency of invasive ventilation (87.5%) in comparison with patients with severe COVID-19 who survived (23.5%) (*p* = 0.007). Furthermore, patients with severe COVID-19 who died stayed a higher number of days in the ICU in comparison with patients with severe COVID-19 who recovered (14.7 ± 6.7 *vs* 4.2 ± 3.1) (*p* = 0.012).

**Figure 3:**
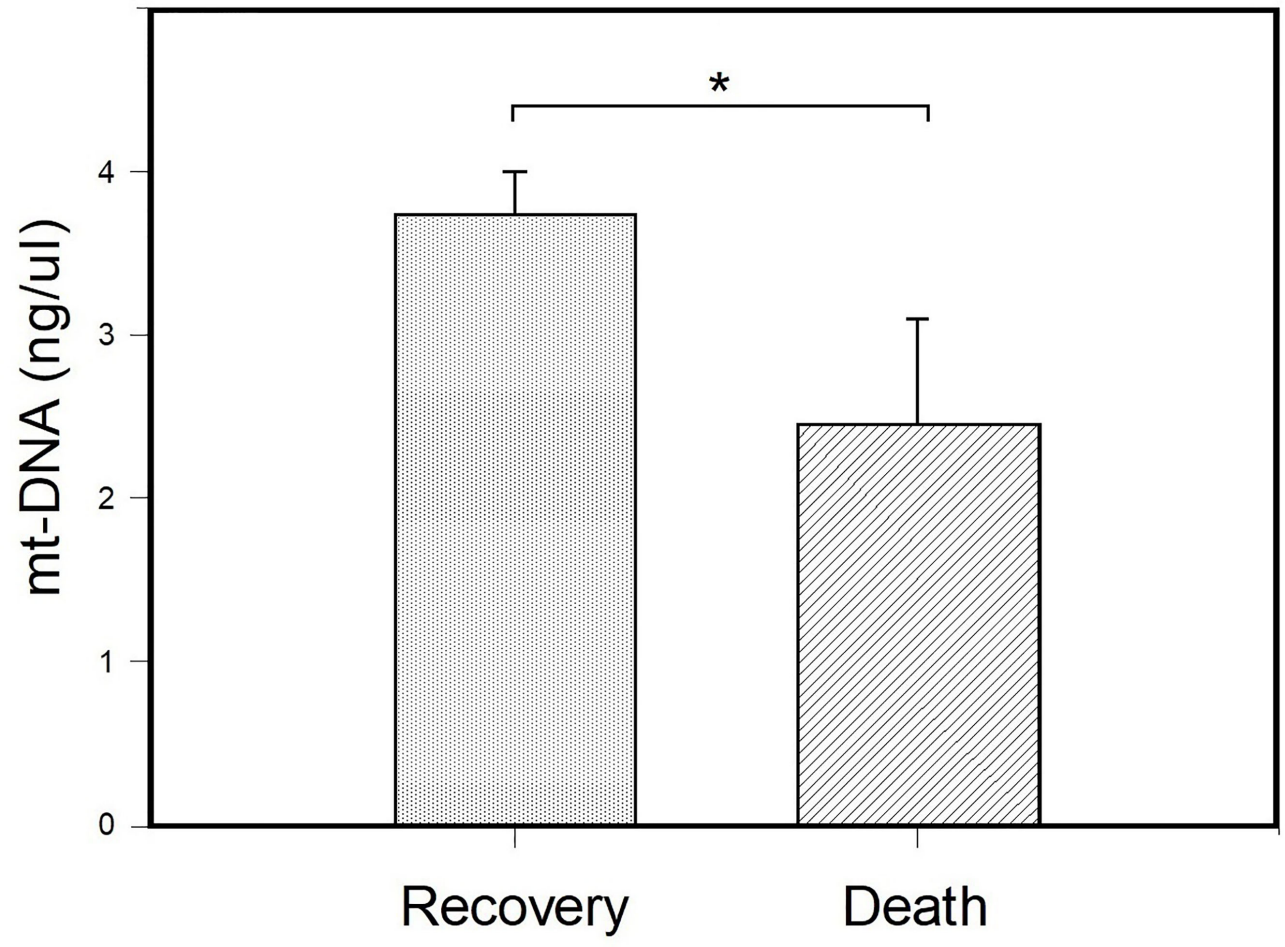
Comparison of peripheral blood mt-DNA between patients with the severe COVID-19 sub- stratified according to their outcome. Patients were classified as patients with COVID-19 who died (n = 8) and those who recovered (n = 17) and their peripheral mt-DNA concentrations (ng/μl) were compared. Bar plots represent mt-DNA mean ± standard error. *p < 0.05 (Student’s t-test).

The results of the comparison of the mt-DNA of patients with severe COVID-19 classified according to their outcome are shown in **Figure 3**. Peripheral mt-DNA levels in hospitalized patients who died were lower (2.44 ± 0.65) than that observed in the mt-DNA of patients with COVID-19 who recovered (3.74 ± 0.26) (*p* = 0.037).

### Diagnostic Ability of mt-DNA as Classifier of Controls and COVID-19 Patients: ROC Analysis

The AUC value derived from the ROC analysis to differentiate non-severe COVID-19 using the peripheral mt-DNA concentrations of the control group and patients with mild to moderate COVID-19, was 0.7632 (95% CI: 0.64 - 0.88; *p* ≤ 0.001). Considering a cutoff value of 3.617 ng/µl (2.02 × 10^8^ number of mt-DNA copies), the diagnostic method achieved a sensitivity 84.6% and a specificity of 62.7%. In the same sense, patients with more than 3.617 ng/μl of peripheral blood mt-DNA showed an increased risk of having mild to moderate COVID-19 (OR = 9.26, 95% CI = 2.77 - 30.98; *p*= 3.0 × 10^−4^). Additional ROC analyses obtained to evaluate the classificatory ability of mt-DNA concentrations of possible exacerbating factors for the evolution of the severity of COVID-19 disease had no statistical significance. These analyses included the following: AUC from peripheral blood mt-DNA concentration of patients with severe COVID-19 stratified in relation to the presence or absence of obesity (AUC = 0.4423, 95% CI: 0.20 to 0.68; *p =* 1.37, cut-off = 3.591, sensitivity = 58%, specificity = 69%), and AUC comparing mt-DNA levels of patients with severe COVID-19 who died and recovered (AUC = 0.3235, 95% CI: 0.074 to 0.57; *p=* 1.83, cut-off = 3.543, sensitivity = 47%, specificity = 50%).

## Discussion

In this study, we aimed to evaluate the utility of mt-DNA as a variable associated with COVID-19 and the severity of patients with this disease. The establishment of mt-DNA relevance as a prognostic biomarker in the outcome of COVID-19 was also tested.

Our results showed that the presence of comorbidities, such as obesity and arterial hypertension were the only risk factors with statistically significant differences between the study groups and variables such as BMI and SBP correlated with mt-DNA levels. Growing evidence has postulated that the pathological and biochemical features produced by the COVID-19 acute inflammatory response could be associated with hypermetabolic states, such as hyperglycemia and obesity (Lahera et al., 2017). These conditions lead to cellular alterations, such as mitochondrial dysfunction, due to its multiple cell and metabolic functions (e.g., energy productions and cellular respiration) (Benard et al., 2007). The high frequency of obesity in the patients with COVID-19 in our study, especially in those with severe COVID-19 when compared with the control group, and the inverse correlation between mt-DNA and BMI highlights the importance of obesity in the evolution and exacerbation of COVID-19 and in mitochondrial malfunction. It is well accepted that obesity alters immunity by modifying the response of cytokines and decreases the cytotoxic cell response of immunocompetent cells, which have an important anti-viral role (Hussain et al., 2020). In addition, obesity disturbs endocrine hormones, such as leptin, and leads to the increase of adipose tissue-specific molecules, called adipokines, generating an altered immunity that favors the intensification of the SARS-CoV-2 infection and exacerbation of the mitochondrial damage (Saleh et al., 2020). The high frequency of obesity and mitochondrial dysfunction caused by COVID-19, reflected as the inverse relation between BMI and the decreased amount of mt-DNA in our results, are similar to those reported by Morandi et al. (2020), in which they associated the decrease of mt-DNA in patients with obesity with mitochondrial dysfunction typical of obesity, assuming a relation between function and tissue mitochondrial content with circulating mt-DNA.

Besides the hypermetabolic response and cell disruption caused by COVID-19, SARS-CoV-2 hijacks the mitochondria for its replication, causing an alteration in its permeability and a leakage of mt-DNA into the circulation (Saleh et al., 2020; Valdes-Aguayo et al., 2021), promoting the activation of the immune system that increases the inflammatory response. Additionally, in patients with severe COVID-19, the inflammation upsurgence (Gallagher, 2001), increased of metabolic alteration (Sin et al., 2020) and continuous mitochondrial dysfunction cause a decrease in mt-DNA production, generating a decrease in circulating mt-DNA. In our results, this can be explained with the decline of peripheral mt-DNA in patients with severe COVID-19 in comparison with patients with mild and moderate COVID-19. Similar results were reported by Marit Wiersma et al. (2020), in which they describe the association of cell-free circulating mt-DNA levels in the serum of patients with atrial fibrillation (AF). Their results showed that circulating mt-DNA levels were increased in early stages of AF but decreased in end-stage or longstanding-persistent AF. Although Marit Wiersma et al. study and ours are not completely comparable because the origin of the sample from which mt-DNA was obtained (cell-free mt-DNA *vs* whole blood mt-DNA), our similarities (increased mt-DNA levels in early stages but decreased in end-stage) can be explained because, differently from nuclear DNA, mt-DNA is vulnerable to ROS damage (Yakes and Van Houten, 1997) released during an inflammatory response, resulting from the lack of effective mt-DNA repair and lack of histone protection (Croteau et al., 1999). Once the mt-DNA is damaged, the levels of mt-DNA are altered and decreased, causing more mitochondrial dysfunction and an exacerbation of the disease in a positive feedback cycle (Dai et al., 2012; Ashar et al., 2017). Therefore, the detection of a decrease in mt-DNA concentration as the severity deteriorates the health of the patient may reflect the mitochondrial and mt-DNA damage caused by the COVID-19 inflammatory response and OS exacerbation and/or because changes of cell type changes in the blood in severe COVID-19 (Files et al., 2021).

The mitochondrial disruption and consecutive decrease in mt-DNA has also been explored in other cardiovascular diseases, such as coronary vascular disease, ischemic heart failure and cardiomyopathies. These studies have described that patients with cardiovascular disease had a lower number of copies of mt-DNA when compared with the control group, confirming that the cardiovascular risk increased as the mt-DNA level diminished (Chen et al., 2014; Huang et al., 2016; Ashar et al., 2017; Liu et al., 2017; Zhang et al., 2017). Similarly, Knez et al. recruited 13 patients with end-stage dilated cardiomyopathy who were hospitalized for worsening of heart failure at the University Medical Centre in Slovenia, finding that lower myocardial mt-DNA content correlated with worse left ventricle function and cell energy depletion, which severely depressed cardiac performance. This would eventually cause insufficient systemic perfusion and an increase of systemic OS, which is reflected by a decrease in peripheral blood mt-DNA content, correlating to that of the myocardium (Ahuja et al., 2013). In our study, the differences obtained when comparing the mt-DNA of patients with severe COVID-19 that died during hospitalization and that observed in patients who had clinical recovery may be related to the limited repair capacity of mt-DNA and its vulnerable structure to ROS (Li et al., 2013) produced by pro-inflammatory cytokines, such as TNF-α, IFN-γ, IL-1, IL-6 and IL-8 released during a severe COVID-19 infection (Li et al., 2013), that concurrently produce intracellular OS and more mitochondrial disruption, triggering intracellular cascades that modify mitochondrial metabolism, OXPHOS and ATP production (Jo et al., 2016). These findings agreed with what was reported by Jaan-Yeh Jeng et al. (2008), they explained that the mt-DNA copy number is not a direct index of mt-DNA damage; instead, it is related to mitochondrial enzyme activity and ATP production, so it can indirectly reflect the function of the mitochondria. Therefore, our results support the use of peripheral blood mt-DNA as a biomarker of mitochondrial function useful to determine the severity and prognosis of patients with COVID-19.

Although the decreased of circulating mt-DNA has been detected in this and previous studies, research as Scozzi et al. study (Scozzi et al., 2021) reported that COVID-19 patients with high cell-free mt-DNA levels are more likely to require admission to ICU and intubation, increasing their risk of death. In this study, they evaluated 97 COVID-19 positive patients, of which 25.8% required intubation and mechanical ventilation, and 56.7% required ICU admission. Cell-free plasma mt-DNA levels were found elevated in patients with COVID-19 and those who required ICU admission and advanced mechanical ventilation in comparison with patients who survived. The apparent differences between studies may be explained because the time in which the sample was taken (diagnosis *vs* after treatment or during intubation or mechanical ventilation *vs* ICU admission, etc.) and the origin of the biological sample from which the mt-DNA was quantified (plasma *vs* whole blood). Mitochondrial dysfunction and variations in mt-DNA levels continue to be explored as an essential factor that is associated with morbidity and mortality in patients infected with SARS-CoV-2, and therefore will be important to perform longitudinal studies of mt-DNA quantification in blood in mild *vs* severe COVID-19 patients to explain the origin of mt-DNA fluctuations through the time and evolution of disease.

## Conclusion

Our study confirmed lower levels of whole blood mt-DNA in patients with severe COVID-19 who had de comorbidities of obesity and arterial hypertension in comparison with control group. The inverse correlation found between whole blood mt-DNA concentration and BMI in patients with severe COVID-19 patients, highlight the importance of these comorbidities in the exacerbation of the disease. Furthermore, the decreased levels of peripheral blood mt-DNA in patients with severe COVID-19 in comparison with patients with mild and moderate COVID-19, and in patients who died when compared with patients with COVID-19 who recovered from disease reflects the importance of the dysfunctional mitochondria in the pathology of COVID-19. Although follow-up and additional studies are required to corroborate our findings, our study support the use of peripheral blood mt-DNA as a potential biomarker to establish a prognosis of severity and mortality in patients with COVID-19, according to the mitochondrial functionality of each individual.

## Data Availability Statement

The raw data supporting the conclusions of this article will be made available by the authors, without undue reservation.

## Ethics Statement

The studies involving human participants were reviewed and approved by Research Committee of the Academic Unit of Human Medicine and Health Sciences from Universidad Autónoma de Zacatecas “Francisco García Salinas” and the Ethic and Investigation Committee of the Hospital General de Zacatecas “Luz González Cosío” (ID number: 0223/2021/C). The patients/participants provided their written informed consent to participate in this study.

## Author Contributions

Conceptualization, MM-F, JV-A and JV-R. Methodology, IG-V and LA-C. Validation, MM-F and SB-S. Formal Analysis, MM-V, MM-F, and CG-F. Investigation, JV-A. Resources, MM-F. Writing – Original Draft Preparation, JV-A, MM-F, LA-C, and JV-R. Writing – Review and& Editing, JV-A and MM-F. Visualization, MM-F, AC-G, DA-H, and IG-V. Supervision, CG-F, IR-S, ID-E, and LA-C. Project Administration, MM-F. All authors have read and agreed to the published version of the manuscript.

## Funding

This work was funded in part by the Programa de Apoyos para el Fortalecimiento de Capacidades para el Diagnostico de COVID-19-CONACYT, with Grant ID: 314340 (A12 xii. 2020) to MM-F. The edition costs were covered by the Academic Unit of Human Medicine and Health Sciences of Universidad Autónoma de Zacatecas.

## Conflict of Interest

The authors declare that the research was conducted in the absence of any commercial or financial relationships that could be construed as a potential conflict of interest.

## Publisher’s Note

All claims expressed in this article are solely those of the authors and do not necessarily represent those of their affiliated organizations, or those of the publisher, the editors and the reviewers. Any product that may be evaluated in this article, or claim that may be made by its manufacturer, is not guaranteed or endorsed by the publisher.

## References

[B1] Ahuja P. Wanagat J. Wang Z. Wang Y. Liem D. A. Ping P.. (2013). Divergent Mitochondrial Biogenesis Responses in Human Cardiomyopathy. Circulation 127 (19), 1957–1967. doi: 10.1161/CIRCULATIONAHA.112.001219 23589024 PMC4236313

[B2] Annesley S. J. Fisher P. R. (2019). Mitochondria in Health and Disease. Cells 8 (7), 1–7. doi: 10.3390/cells8070680 PMC6678092 31284394

[B3] Ashar F. N. Zhang Y. Longchamps R. J. Lane J. Moes A. Grove M. L.. (2017). Association of Mitochondrial DNA Copy Number With Cardiovascular Disease. JAMA Cardiol. 2 (11), 1247–1255. doi: 10.1001/jamacardio.2017.3683 29049454 PMC5710361

[B4] Benard G. Bellance N. James D. Parrone P. Fernandez H. Letellier T.. (2007). Mitochondrial Bioenergetics and Structural Network Organization. J. Cell Sci. 120 (Pt 5), 838–848. doi: 10.1242/jcs.03381 17298981

[B5] Cheng H. Y. Jian S. W. Liu D. P. Ng T. C. Huang W. T. Lin H. H.. (2020). Contact Tracing Assessment of COVID-19 Transmission Dynamics in Taiwan and Risk at Different Exposure Periods Before and After Symptom Onset. JAMA Intern. Med. 180 (9), 1156–1163. doi: 10.1001/jamainternmed.2020.2020 32356867 PMC7195694

[B6] Chen S. Xie X. Wang Y. Gao Y. Xie X. Yang J.. (2014). Association Between Leukocyte Mitochondrial DNA Content and Risk of Coronary Heart Disease: A Case-Control Study. Atherosclerosis 237 (1), 220–226. doi: 10.1016/j.atherosclerosis.2014.08.051 25244506

[B7] Croteau D. L. Stierum R. H. Bohr V. A. (1999). Mitochondrial DNA Repair Pathways. Mutat. Res. 434 (3), 137–148. doi: 10.1016/s0921-8777(99)00025-7 10486588

[B8] Dai D. F. Rabinovitch P. S. Ungvari Z. (2012). Mitochondria and Cardiovascular Aging. Circ. Res. 110 (8), 1109–1124. doi: 10.1161/CIRCRESAHA.111.246140 22499901 PMC3867977

[B9] Dong E. Du H. Gardner L. (2020). An Interactive Web-Based Dashboard to Track COVID-19 in Real Time. Lancet Infect. Dis. 20 (5), 533–534. doi: 10.1016/S1473-3099(20)30120-1 32087114 PMC7159018

[B10] Faas M. M. de Vos P. (2020). Mitochondrial Function in Immune Cells in Health and Disease. Biochim. Biophys. Acta Mol. Basis Dis. 1866 (10):165845. doi: 10.1016/j.bbadis.2020.165845 32473386

[B11] Files J. K. Boppana S. Perez M. D. Sarkar S. Lowman K. E. Qin K.. (2021). Sustained Cellular Immune Dysregulation in Individuals Recovering From SARS-CoV-2 Infection. J. Clin. Invest. 131 (1), 1–29. doi: 10.1172/JCI140491 PMC7773371 33119547

[B12] Gallagher TM, B.M. (2001). Coronavirus Spike Proteins in Viral Entry and Pathogenesis. Virology 279 (2), 371–374. doi: 10.1006/viro.2000.0757 11162792 PMC7133764

[B13] Hagemeijer M. C. V. A. Monastyrska I. Rottier P. J. de Haan C. A. (2012). Visualizing Coronavirus RNA Synthesis in Time by Using Click Chemistry. Virol 86, 5808–5816. doi: 10.1128/JVI.07207-11 PMC3347275 22438542

[B14] Hamming I. van Goor H Turner A. J. Rushworth C. A. Michaud A. A. Corvol P.. (2008). Differential Regulation of Renal Angiotensin-Converting Enzyme (ACE) and ACE2 During ACE Inhibition and Dietary Sodium Restriction in Healthy Rats. Exp. Physiol. 93, 631–638. doi: 10.1113/expphysiol.2007.041855 18192334

[B15] Hoffmann M. K.-W H. Schroeder S. Krüger N. Herrler T. Erichsen S.. (2020). SARS-CoV-2 Cell Entry Depends on ACE2 and TMPRSS2 and Is Blocked by a Clinically Proven Protease Inhibitor. Cell 181, 271–280. doi: 10.1016/j.cell.2020.02.052 32142651 PMC7102627

[B16] Huang J. Tan L. Shen R. Zhang L. Zuo H. Wang D. W. (2016). Decreased Peripheral Mitochondrial DNA Copy Number Is Associated With the Risk of Heart Failure and Long-Term Outcomes. Med. (Baltimore) 95 (15), e3323. doi: 10.1097/MD.0000000000003323 PMC4839823 27082579

[B17] Hussain A. Mahawar K. Xia Z. Yang W. El-Hasani S. (2020). Obesity and Mortality of COVID-19. Meta-Analysis. Obes. Res. Clin. Pract. 14 (4), 295–300. doi: 10.1016/j.orcp.2020.07.002 32660813 PMC7346803

[B18] Información, I.N.d.E.G.e (2020). Mortalidad Conjunto De Datos: Defunciones Por Homicidios (Mexico City: INEGI).

[B19] Jeng J. Y. Yeh T. S. Lee J. W. Lin S. H. Fong T. H. Hsieh R. H. (2008). Maintenance of Mitochondrial DNA Copy Number and Expression Are Essential for Preservation of Mitochondrial Function and Cell Growth. J. Cell Biochem. 103 (2), 347–357. doi: 10.1002/jcb.21625 18072287

[B20] Jo E. K. Kim J. K. Shin D. M. Sasakawa C. (2016). Molecular Mechanisms Regulating NLRP3 Inflammasome Activation. Cell Mol. Immunol. 13 (2), 148–159. doi: 10.1038/cmi.2015.95 26549800 PMC4786634

[B21] King A. M. Q Adams M. J Carstens E. B. Lefkowitz E. J.. (2012). Ninth Report of the International Committee on Taxonomy of Viruses. Elsevier 770–783.

[B22] Knoops K. Kikkert M. Worm S. H. Zevenhoven-Dobbe J. C. van der Meer Y. Koster A. J.. (2008). SARS-Coronavirus Replication Is Supported by a Reticulovesicular Network of Modified Endoplasmic Reticulum. PloS Biol. 6, 226. doi: 10.1371/journal.pbio.0060226 PMC2535663 18798692

[B23] Lahera V. de Las Heras N. Lopez-Farre A. Manucha W. Ferder L. (2017). Role of Mitochondrial Dysfunction in Hypertension and Obesity. Curr. Hypertens. Rep. 19 (2), 11. doi: 10.1007/s11906-017-0710-9 28233236

[B24] Liang W. Liang H. Ou L. Chen B. Chen A. Li C.. (2020). Development and Validation of a Clinical Risk Score to Predict the Occurrence of Critical Illness in Hospitalized Patients With COVID-19. JAMA Intern. Med. 180 (8), 1081–1089. doi: 10.1001/jamainternmed.2020.2033 32396163 PMC7218676

[B25] Liao P. C. Bergamini C. Fato R. Pon L. A. Pallotti F. (2020). Isolation of Mitochondria From Cells and Tissues. Methods Cell Biol. 155, 3–31. doi: 10.1016/bs.mcb.2019.10.002 32183964 PMC8530414

[B26] Li X. Fang P. Mai J. Choi E. T. Wang H. Yang X. F. (2013). Targeting Mitochondrial Reactive Oxygen Species as Novel Therapy for Inflammatory Diseases and Cancers. J. Hematol. Oncol. 6, 19. doi: 10.1186/1756-8722-6-19 23442817 PMC3599349

[B27] Liu L. P. Cheng K. Ning M. A. Li H. H. Wang H. C. Li F.. (2017). Association Between Peripheral Blood Cells Mitochondrial DNA Content and Severity of Coronary Heart Disease. Atherosclerosis 261, 105–110. doi: 10.1016/j.atherosclerosis.2017.02.013 28242046

[B28] Lowes H. Pyle A. Santibanez-Koref M. Hudson G. (2020). Circulating Cell-Free Mitochondrial DNA Levels in Parkinson's Disease Are Influenced by Treatment. Mol. Neurodegener. 15 (1), 10. doi: 10.1186/s13024-020-00362-y 32070373 PMC7029508

[B29] Mejia-Vilet J. M. Cordova-Sanchez B. M. Fernandez-Camargo D. A. Mendez-Perez R. A. Morales-Buenrostro L. E. Hernandez-Gilsoul T. (2020). A Risk Score to Predict Admission to the Intensive Care Unit in Patients With COVID-19: The ABC-GOALS Score. Salud Publica Mex 63 (1, ene-feb), 1–11. doi: 10.21149/11684 33021362

[B30] Morandi A. Zusi C. Corradi M. Takemoto K. Contreas G. Olivieri F.. (2020). Circulating Mitochondrial DNA Is Decreased in Children and Adolescents With Obesity and/or Insulin Resistance. Acta Diabetol. 57 (5), 623–625. doi: 10.1007/s00592-019-01471-x 31953686

[B31] Nakahira K. Kyung S. Y. Rogers A. J. Gazourian L. Youn S. Massaro A. F.. (2013). Circulating Mitochondrial DNA in Patients in the ICU as a Marker of Mortality: Derivation and Validation. PloS Med. 10 (12), e1001577; discussion e1001577. doi: 10.1371/journal.pmed.1001577 24391478 PMC3876981

[B32] Nunnari J. Suomalainen A. (2012). Mitochondria: In Sickness and in Health. Cell 148 (6), 1145–1159. doi: 10.1016/j.cell.2012.02.035 22424226 PMC5381524

[B33] Saleh J. P. Carole Keshav K.S. Marvin E. (2020). Mitochondria and Microbiota Dysfunction in COVID-19 Pathogenesis. Elsevier 54, 1–7. doi: 10.1016/j.mito.2020.06.008 PMC7837003 32574708

[B34] Scozzi D. Cano M. Ma L. Zhou D. Zhu J. H. O'Halloran J. A.. (2021). Circulating Mitochondrial DNA Is an Early Indicator of Severe Illness and Mortality From COVID-19. JCI Insight 6 (4), 1–16. doi: 10.1172/jci.insight.143299 PMC7934921 33444289

[B35] Secretaría de Salud. (2020). “Lineamiento Para La Atención De Paciente Por COVID-19,” in Secretaría De Salud, Comisión Coordinadora De Institutos Nacionales De Salud Y Hospitales De Alta Especilidad (Mexico City: Secretia de Salud).

[B36] Sethna P. B. Hung S. L. Brian D. A. (1989). Coronavirus Subgenomic Minus-Strand RNAs and the Potential for mRNA Replicons. Proc. Natl. Acad. Sci. U. S. A. 86 (14), 5626–5630. doi: 10.1073/pnas.86.14.5626 2546161 PMC297677

[B37] Sharma P. Sampath H. (2019). Mitochondrial DNA Integrity: Role in Health and Disease. Cells 8 (2), 1–21. doi: 10.3390/cells8020100 PMC6406942 30700008

[B38] Sharma A. Schaefer S. T. Sae-Lee C. Byun H. M. Wullner U. (2021). Elevated Serum Mitochondrial DNA in Females and Lack of Altered Platelet Mitochondrial Methylation in Patients With Parkinson s Disease. Int. J. Neurosci. 131 (3), 279–282. doi: 10.1080/00207454.2020.1738433 32125208

[B39] Shenoy S. (2020). Coronavirus (Covid-19) Sepsis: Revisiting Mitochondrial Dysfunction in Pathogenesis, Aging, Inflammation, and Mortality. Inflammation Res. 69 (11), 1077–1085. doi: 10.1007/s00011-020-01389-z PMC7410962 32767095

[B40] Singer M. (2014). The Role of Mitochondrial Dysfunction in Sepsisinduced Multi-Organ Failure. Virulence 5, 66–72. doi: 10.4161/viru.26907 24185508 PMC3916385

[B41] Sin R. Kubiska M. Cmorej P. C. Vachalova J. (2020). Clinical and Laboratory Characteristics of the COVID-19 Disease in Adult Patients. Neuro Endocrinol. Lett. 41 (5), 223–230. 33315336

[B42] Valdes-Aguayo J. J. Garza-Veloz I. Badillo-Almaraz J. I. Bernal-Silva S. Martinez-Vazquez M. C. Juarez-Alcala V.. (2021). Mitochondria and Mitochondrial DNA: Key Elements in the Pathogenesis and Exacerbation of the Inflammatory State Caused by COVID-19. Med (Kaunas) 57 (9), 1–22. doi: 10.3390/medicina57090928 PMC8471487 34577851

[B43] Wiersma M. van Marion D. M. S. Bouman E. J. Li J. Zhang D. Ramos K. S.. (2020). Cell-Free Circulating Mitochondrial DNA: A Potential Blood-Based Marker for Atrial Fibrillation. Cells 9 (5), 1–16. doi: 10.3390/cells9051159 PMC7290331 32397106

[B44] Yakes F. M. Van Houten B. (1997). Mitochondrial DNA Damage Is More Extensive and Persists Longer Than Nuclear DNA Damage in Human Cells Following Oxidative Stress. Proc. Natl. Acad. Sci. U. S. A. 94 (2), 514–519. doi: 10.1073/pnas.94.2.514 9012815 PMC19544

[B45] Yue P. Jing S. Liu L. Ma F. Zhang Y. Wang C.. (2018). Association Between Mitochondrial DNA Copy Number and Cardiovascular Disease: Current Evidence Based on a Systematic Review and Meta-Analysis. PloS One 13 (11), e0206003. doi: 10.1371/journal.pone.0206003 30403687 PMC6221293

[B46] Zhang Y. Guallar E. Ashar F. N. Longchamps R. J. Castellani C. A. Lane J.. (2017). Association Between Mitochondrial DNA Copy Number and Sudden Cardiac Death: Findings From the Atherosclerosis Risk in Communities Study (ARIC). Eur. Heart J. 38 (46), 3443–3448. doi: 10.1093/eurheartj/ehx354 29020391 PMC5837579

[B47] Zhou P. Yang X. L. Wang X. G. Hu B. Zhang L. Zhang W.. (2020). A Pneumonia Outbreak Associated With a New Coronavirus of Probable Bat Origin. Nature 579 (7798), 270–273. doi: 10.1038/s41586-020-2012-7 32015507 PMC7095418

